# Genotype and Phenotype Description in Individuals With *GYG1* Variants—A Case Report and Systematic Review

**DOI:** 10.1002/jmd2.70134

**Published:** 2026-11-05

**Authors:** M. Schadeck, A. Tankeu, J. Elmers, E. Hewer, F. Maurer, P. Monney, M. Théaudin, C. Tran

**Affiliations:** ^1^ Division of Genetic Medicine University Hospital of Lausanne (CHUV) and University of Lausanne (UNIL) Lausanne Switzerland; ^2^ Service of Endocrinology, Diabetes and Metabolism University Hospital of Lausanne (CHUV) and University of Lausanne (UNIL) Lausanne Switzerland; ^3^ Medical Library University Hospital of Lausanne (CHUV) and University of Lausanne (UNIL) Lausanne Switzerland; ^4^ Institute of Pathology University Hospital of Lausanne (CHUV) and University of Lausanne (UNIL) Lausanne Switzerland; ^5^ Department of Cardiology University Hospital of Lausanne (CHUV) and University of Lausanne (UNIL) Lausanne Switzerland; ^6^ Department of Clinical Neurosciences, Service of Neurology, Nerve‐Muscle Unit University Hospital of Lausanne (CHUV) and University of Lausanne (UNIL) Lausanne Switzerland

**Keywords:** cardiomyopathy, genotype–phenotype correlation, glycogen storage disease, *GYG1*, myopathy, systematic review

## Abstract

Glycogen Storage Disease Type XV (GSD XV) is a rare autosomal recessive disorder caused by biallelic pathogenic variants in *GYG1*, encoding glycogenin‐1. Although primarily a myopathic disease, cardiac involvement is increasingly recognized and overlapping features with other neuromuscular disorders contribute to underdiagnosis. We performed a systematic search of databases without restrictions on publication dates. Twenty‐one articles describing individuals with GSD XV were included, providing clinical, histological, and genetic data. In addition to the systematic review, we present an illustrative case of GSD XV diagnosed at our institution. We identified 49 individuals, of whom 98% were symptomatic with onset ranging from childhood to late adulthood; manifestations were isolated neuromuscular (81.6%), cardiac (8.2%), or mixed (8.2%). One case (2.0%) was asymptomatic. Among neuromuscular cases, 12% required a walking aid, while four cardiac patients required implantable cardioverter‐defibrillators and two underwent heart transplantation. Muscle biopsy frequently revealed PAS‐positive, diastase‐resistant polyglucosan bodies, although polyglucosan bodies were not consistently identified by electron microscopy. Molecular diagnosis was confirmed in all cases, with c.143+3G>C in *GYG1* being the most prevalent variant, homozygous in 36.7% of patients and associated with both juvenile and adult‐onset phenotypes. GSD XV should be considered in cases with unexplained myopathy or cardiomyopathy, regardless of age at onset. Genetic testing remains the gold standard for diagnosis, although muscle biopsy can guide investigations, particularly in atypical cases or when genetic analysis is inconclusive. Further studies are needed to clarify the full clinical spectrum, refine genotype–phenotype correlations, and guide long‐term management strategies.

## Synopsis

Glycogen Storage Disease Type XV is an underrecognized disorder with a broad neuromuscular and cardiac spectrum across all ages, in which genetic testing is essential for diagnosis and should be considered even when muscle biopsy findings are inconclusive.

## 1 Introduction

Glycogen storage diseases (GSD) are inherited disorders of metabolism affecting glycogen synthesis or degradation of glycogen mostly in the liver and muscles. Over the last 7 decades, since the first description of GSD Type I in 1929 by von Gierke [1], 14 other types have been discovered. GSD Type XV (polyglucosan body myopathy‐2 [PGBM‐2 OMIM #616199]) was first described in 2010 by Moslemi et al. and is caused by biallelic loss‐of‐function mutations in the *GYG1* gene [2, 3]. This gene is located on chromosome 3q24 and encodes glycogenin‐1, a glycosyltransferase that generates, by autoglycosylation, a short glucose polymer of approximately 10 glucose residues. This primer is then elongated and branched by glycogen synthase and branching enzyme [4, 5]. GYG1 is expressed in both muscle and heart, and its deficiency disrupts the initiation of glycogen synthesis, leading to impaired energy storage in these tissues [2, 6, 7]. This results in glycogen depletion and abnormal glycogen accumulation, histologically seen as polyglucosan inclusions. Polyglucosan is a poorly branched polysaccharide that forms insoluble aggregates, which are PAS‐positive but diastase‐resistant. In GYG1 deficiency, residual glycogen synthesis may occur via glycogenin‐independent mechanisms, producing structurally abnormal glycogen that accumulates as polyglucosan bodies [8].

Most cases present with progressive weakness of proximal and/or distal muscles, sometimes mimicking limb‐girdle muscle dystrophy [9, 10]. Rarely, the only presentation is a dilated or hypertrophic cardiomyopathy, and only a few cases combine both cardiomyopathy and skeletal myopathy [3, 11, 12]. Individuals exhibit a wide range of ages of onset, from early childhood to mid‐70s, suggesting that other genetic, epigenetic, or environmental factors may influence disease expression.

Histological examination typically reveals PAS‐positive, diastase‐resistant polyglucosan bodies within muscle fibers, described as aggregations of polysaccharides [3]. Electroneuromyography (ENMG) studies often indicate myopathy with a rare neurogenic pattern [4, 13, 14].

In GSD XV, genotype–phenotype correlation remains poorly defined, and no consistent relationship between specific variants and clinical presentation has been established. The disease is generally considered to result from loss of glycogenin‐1 function due to absent or reduced protein expression [2, 3]. The most common variant, c.143+3G>C (NM_004130.4), is predicted to cause exon 2 skipping, leading to nonsense‐mediated decay or a truncated protein [4, 13]. To date, 48 cases of GSD XV have been reported, including 13 homozygous for this variant. Despite these reports, systematic data on natural history and genotype–phenotype correlations remain limited, likely contributing to underrecognition of the disease.

Here, we present the case of the oldest known patient, at the time of diagnosis, carrying the homozygous c.143+3G>C variant who was affected by insidious progressive muscle weakness since he was 75 years old. In addition, we conducted a systematic review of the literature of 48 cases. We aimed to explore the presenting symptoms, clinical manifestations, histology findings, muscle imaging, cardiac involvement, and genetic analysis.

## 2 Case Report

An 82‐year‐old patient was referred to our department for investigation of progressive lower extremity weakness and unsteady gait that were most pronounced when walking uphill. Symptoms started at age 75 and were slowly progressive in a patient who was otherwise independent and physically active without any complaints.

The upper body and face were unaffected, the patient had no respiratory complaints, and the thoracic movements were not altered. He did not recall any worsening factors, nor did he associate the beginning of the symptoms with an infection. He reported mild memory loss and a neurocognitive evaluation demonstrated slightly reduced neurocognitive abilities with moderate attention deficit. He noticed a mild hearing loss which has not been investigated. The patient had no signs of rhabdomyolysis, nor pain, numbness, paresthesia, or other sensory symptoms. Motor development and health during childhood were uneventful. The family history was noncontributory.

Clinical evaluation showed atrophy of the right foot muscles. There were fasciculations in the right trapezius and a slight bilateral scapula alata. Motor testing showed a slight proximal tetraparesis and paresis of the extensors digitorum brevis. Tendon reflexes were amplified in the upper body and normal in the lower body. The patient had no ataxia but demonstrated a bilateral Trendelenburg gait. He showed no Gowers sign while getting up and walked without difficulty on tiptoes and heels.

Given this clinical presentation of isolated progressive muscle weakness suggestive of a metabolic myopathy or another neuromuscular disorder, the patient underwent a muscle biopsy, a brain MRI, and cardiac evaluation including ECG, echocardiography, and cardiac MRI.

Muscle biopsy of the left gastrocnemius muscle showed an intact fascicular architecture, no necrotic fibers, no fibrosis, and no inflammatory infiltrate (Figure 1). There was only a very mild increase in fiber size variation. Slight fiber type grouping was identified by ATPase enzyme histochemistry (not shown), consistent with collateral reinnervation indicating a mild neurogenic affection. Rare fibers (< 1%) contained PAS‐positive, diastase‐resistant intracytoplasmic inclusions consistent with polyglucosan bodies (Figure 1).

**FIGURE 1:**
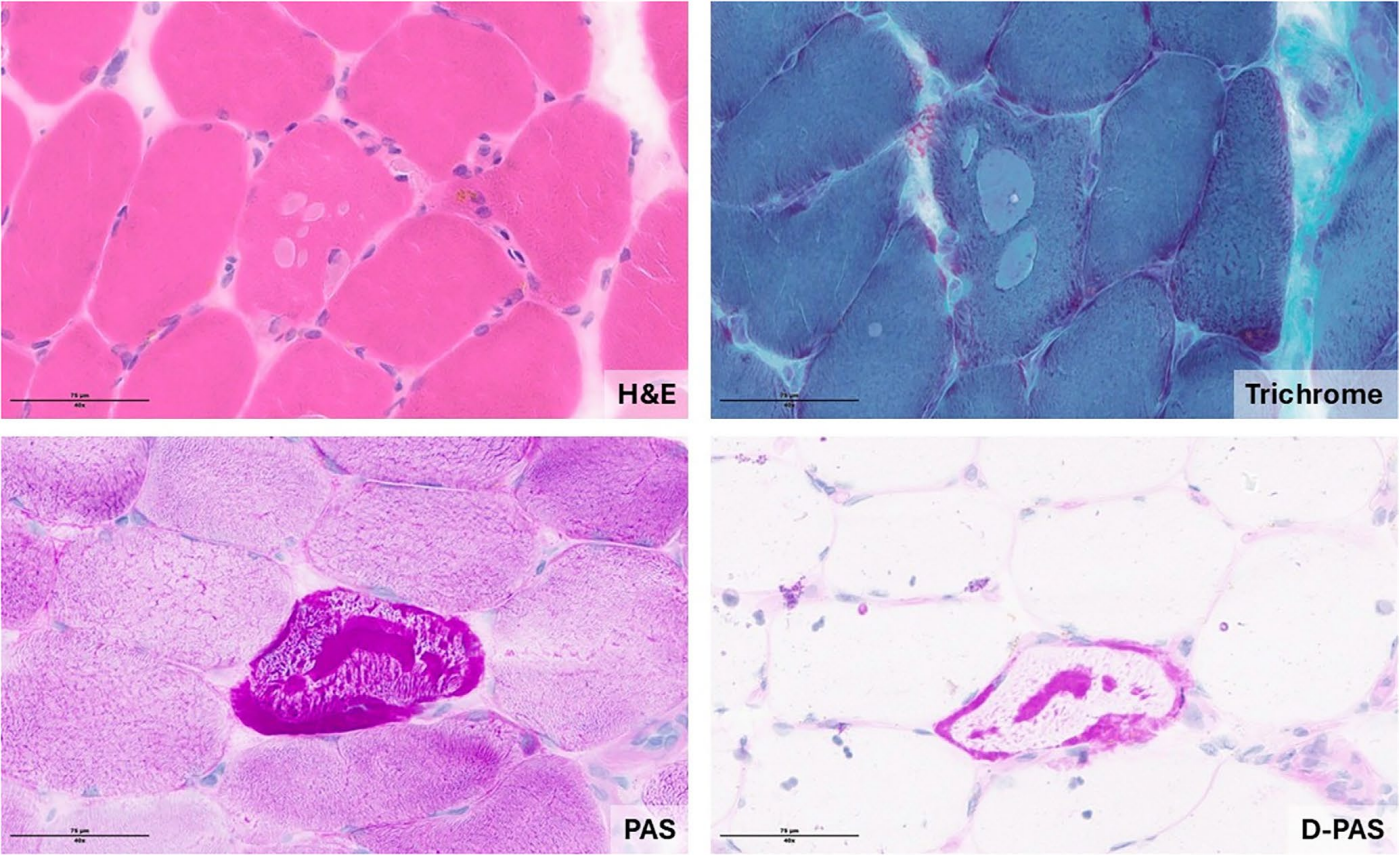
Muscle biopsy of the left gastrocnemius muscle showed an intact fascicular architecture, no necrotic fibers, no fibrosis, and no inflammatory infiltrate. There was only a very mild increase in fiber size variation. Slight fiber type grouping was identified by ATPase enzyme histochemistry (not shown), consistent with collateral reinnervation indicating a mild neurogenic affection. Rare fibers (< 1%) showed spherical or elongated intracytoplasmic inclusions that were pale upon hematoxylin–eosin and Gömöri trichrome staining, positive with periodic‐acid/Schiff's reagent (PAS) staining and resistant to diastase digestion, consistent with polyglucosan bodies. Their diameter varied between 10 and 30 μm.

Brain MRI showed subcortical frontal bilateral and parietal atrophy as well as T2‐hyperintensities compatible with vascular leukoencephalopathy. ECG, echocardiography, and cardiac MRI showed no evidence of cardiomyopathy or myocardial fibrosis. CK was slightly elevated (227 U/L).

Genetic testing of a customized genepanel associated with polyglucosan body myopathies identified the most frequent pathogenic variant in *GYG1* reported to date (NM_004130.4(*GYG1*): c.143+3G>C p.(?)) in a homozygous state.

## 3 Methodology

### 3.1 Search Protocol and Inclusion Criteria

A systematic literature review was conducted according to systematic review methodology and reported following PRISMA guidelines (Preferred Reporting Items for Systematic reviews and Meta‐Analyses, https://www.prisma‐statement.org/). In collaboration with a biomedical information specialist (J.E.) at Lausanne University Medical Library, we devised an overall search strategy and confirmed the search parameters. The search strategy was translated for the following databases: Medline ALL Ovid, Embase.com, Web of Science Core collection, Cochrane Central Register of Controlled Trials Wiley, PubMed Central, Clinicaltrials.gov, ICTRP, and Google Scholar. All searches were conducted on February 28th, 2025.

Two authors (M.S. and A.T.) independently screened the title and abstract of each article. Full‐text articles were then reviewed based on the inclusion criteria using Rayyan [10]. Any inconsistencies were discussed and agreement reached. We specifically included articles reporting individuals with genetically confirmed glycogenosis Type XV and their clinical presentation. Abstracts, poster presentations, and review articles that did not include original human and clinical research articles were not included.

### 3.2 Data Extraction

The following data were extracted from the articles: age at diagnosis, age of symptom onset, presenting symptoms, histology findings, electron microscopy (EM), ENMG/EMG, muscle imaging, CK levels, cardiac evaluation, and genetic results.

We used descriptive statistics to summarize quantitative data while qualitatively describing clinical manifestations. The means and percentages were calculated using Microsoft 365 Excel Version 2507. Data were rounded to one decimal. The protocol of this systematic study was pre‐registered in the NIHR International Prospective Register of Systematic Reviews (https://www.crd.york.ac.uk/PROSPERO/view/CRD42025602895).

### 3.3 Muscle Weakness Assessment

Muscle weakness was assessed clinically using the Medical Research Council (MRC) scale (specific scores not extracted) or by patient report and categorized by distribution and pattern.

Muscle volume was classified as atrophic, hypertrophic, or mixed. Additional features were extracted when reported. When data were not specified, absence could not be distinguished from missing information; these cases were grouped together. Use of walking aids was recorded when available.

### 3.4 Cardiac Involvement

Cardiac manifestations were classified as absent or present and further subcategorized into cardiomyopathy, arrhythmia, ischemic heart disease, valvular disease, and conduction defects. Cardiomyopathy encompassed hypertrophic and dilated forms. Data on cardiac interventions, including implantable cardioverter‐defibrillator (ICD) and heart transplantation, were collected when available.

### 3.5 Histopathological Analysis

Biopsy data from skeletal muscle and/or myocardium were reviewed. Histological techniques included light microscopy with periodic acid–Schiff (PAS) staining, with or without diastase digestion, and EM.

### 3.6 Creatine Kinase (CK) Levels

CK levels were considered elevated if at least one recorded measurement exceeded 140 U/L, regardless of whether other values in the same patient were within the normal range.

### 3.7 Electroneuromyography (ENMG) or electromyography (EMG)

ENMG or EMG findings were categorized as myopathic, neurogenic, or mixed patterns.

### 3.8 Muscle Imaging

Muscle imaging was considered abnormal when fatty replacement, atrophy, degeneration, or myopathic changes were reported.

## 4 Results

### 4.1 Study Selection

This study includes 21 publications reporting 48 individuals with GSD XV in addition to the present case [3, 4, 5, 9, 10, 11, 12, 13, 14, 15, 16, 17, 18, 19, 20, 21, 22, 23, 24, 25, 26, 27]. Supplementary Figure 1 shows the study's flowchart adapted from PRISMA 2020 flow diagram.

### 4.2 Participant Characteristics

We collected a total of 49 cases with a genetically confirmed diagnosis of GSD XV, including the present case. 51% of cases were men, 49% of cases were women (Table 1).

**TABLE 1:** Demographic findings and age of onset.

	Number of patients (%)
Male/female	25/24 (51/49)
*Age of onset*	
0–10 years	1 (2)
11–18 years	6 (12)
19–40 years	13 (27)
41–60 years	17 (35)
61–80 years	7 (14)
> 80 years	1 (2)
Data n.a.	4 (8)
*Age at description*	
0–10 years	0 (0)
11–18 years	0 (0)
19–40 years	9 (18)
41–60 years	12 (24)
61–80 years	22 (45)
> 80 years	5 (10)
Data n.a.	1 (2)

The most frequent category for age of onset was Group 3 (41–60 years) and mean age of onset was 46.8 ± 17.2 years (range 15–75 years) [13, 21], counting all 39 cases with precise age of onset. Ten cases with an estimated age of onset (“childhood,” “teens,” no known age of onset) were not included in this calculation [4, 9, 13, 16, 22]. Seven (14%) individuals were diagnosed during childhood or adolescence (< 18 years).

Mean age at description was 59.6 ± 17.5 years (range 23–84 years).

### 4.3 Muscular Involvement: Symptoms and Signs

At the time of diagnosis, 98% (*n* = 48) cases were symptomatic. One patient was asymptomatic and diagnosed through cascade family screening following a confirmed diagnosis in a family member [13].

The main clinical features are summarized in Table 2 and primarily included muscle weakness and muscle atrophy. The most frequent presenting symptom was muscle weakness, observed in 90% of cases (*n* = 44). Among the five cases without reported muscle involvement, four had cardiomyopathy.

**TABLE 2:** Clinical, biological, electrophysiological, and imaging findings at diagnosis (*n* = 49).

	Number of patients (%)
*Muscle weakness*	
No muscle weakness	5 (10)
Upper limb weakness: proximal	0 (0)
Upper limb weakness: distal	1 (2)
Upper limb weakness proximal and distal	1 (2)
Lower limb weakness: proximal	6 (12)
Lower limb weakness: distal	0 (0)
Lower limb weakness: proximal and distal	6 (12)
Upper and lower limb weakness	30 (61)
Walking aid	6 (12)
No walking aid or data n.a.	43 (88)
Facial weakness	4 (8)
No facial weakness or data n.a.	45 (92)
Scapular winging	15 (31)
No scapular winging or data n.a.	34 (69)
Symmetric distribution	3 (6)
Asymmetric distribution	9 (18)
Symmetric and asymmetric distribution	2 (4)
Symmetric or asymmetric: data n.a.	35 (71)
Muscle atrophy	21 (43)
Muscle hypertrophy	2 (4)
Muscle atrophy and hypertrophy	2 (4)
Atrophy or hypertrophy: data n.a.	24 (49)
Shortness of breath	6 (12)
No shortness of breath or data n.a.	43 (88)
*Cardiac presentation*	
No cardiac involvement	33 (67)
Cardiomyopathy	6 (12)
Isolated arythmia	0 (0)
Cardiomyopathy and arythmia	2 (4)
Ischemic cardiopathy	4 (8)
Valvular dysfunction	3 (6)
Conduction defects	1 (2)
ICD implantation	2 (4)
Heart transplant	2 (4)
No implantable cardioverter‐defibrillator or transplant	45 (94)
*CK‐levels*	
CK‐level > 140 U/L	21 (43)
CK‐level < 140 U/L	20 (41)
CK levels not available	8 (16)
*Biopsy findings*	
No biopsy	5 (10)
Muscle biopsy	39 (80)
Myocardial biopsy	3 (6)
Muscle and myocardial biopsy	2 (4)
No findings in optic microscopy or data n.a.	10 (20)
PAS inclusions	6 (12)
PAS incluisions + alpha‐amylase resistance	33 (67)
No electronic microscopy findings	19 (39)
Polyglucosan bodies in EM	19 (39)
No polyglucosan bodies in EM	11 (22)
*Electroneuromyography/Electromyography*	
No Electroneuromyography/Electromyography or data n.a.	17 (35)
Myopathic pattern	25 (51)
Neurogenic pattern	1 (2)
Myopathic and neurogenic pattern	4 (8)
Normal electromyography	2 (4)
*Imaging*	
No imaging finding reported	25 (51)
Normal MRI	0 (0)
Abnormal MRI	15 (31)
Normal CT	0 (0)
Abnormal CT	1 (2)
Abnormal MRI and CT	4 (8)
Abnormal cardiac MRI	4 (8)

The most common distribution of muscle weakness was both upper and lower limbs (*n* = 31) followed by isolated proximal lower limb weakness (*n* = 5) and combined proximal and distal lower limb weakness (*n* = 6). An asymmetric pattern of muscle weakness was reported in nine cases. A symmetric distribution was documented in our case and two reported cases [11]. Two additional cases showed regional variation in the distribution of weakness, with symmetric involvement in some muscle groups and asymmetric involvement in others [4, 27]. There was no available information regarding lateralization in 35 cases.

When data were available (51%, *n* = 25), muscle atrophy was the second most reported symptom, present in 21 cases with 2 additional cases presenting with muscle hypertrophy and 2 cases combining atrophy and hypertrophy. Scapular winging was observed in 31% of the patients (*n* = 15) and facial muscle weakness in 8% (*n* = 4) of cases.

Walking aids were reported in 6 cases with 49 years being the youngest patient and 74 years the oldest patient [13, 20]. The other cases required walking aids at 59 and 60 years [15, 17]. Five of these cases carried the homozygous c.143 + 3G>C mutation.

### 4.4 Diagnosis of Individuals With GSDXV

#### 4.4.1 ENMG/EMG

ENMG/EMG data were available for 32 cases (65%, *n* = 32). Among these, 25 cases exhibited a myopathic pattern, 1 a neurogenic pattern, 4 a mixed pattern, and 2 cases had normal EMG findings [5, 20].

#### 4.4.2 Muscle Imaging

Muscle imaging was available in 51% (*n* = 24) of cases and was consistently abnormal when reported. The specific imaging patterns were not further classified, as most descriptions referred to general muscle involvement or fatty replacement.

#### 4.4.3 CK Levels

Serum CK levels were elevated (> 140 U/L) in 41% (*n* = 20) of cases, with values ranging from 147 to 1509 U/L [10, 14], indicating mild to moderate elevation. No cases of rhabdomyolysis were reported. CK levels were within the normal range in 43% of cases, and data were unavailable in 16%.

#### 4.4.4 Histology

Among the 43 cases with available histological data, 33 (75%) showed PAS‐positive, diastase‐resistant inclusions consistent with polyglucosan bodies. Six additional biopsies showed PAS‐positive material, although diastase resistance was not reported, and one case demonstrated PAS‐positive material that was sensitive to diastase digestion [19].

Five biopsies did not demonstrate characteristic findings on EM, although two of these cases were still reported to contain polyglucosan bodies [13].

EM was performed in 30 cases and identified polyglucosan bodies in 19; explicit absence was reported in only one patient described by Akman et al. [14].

### 4.5 Disease Onset in Children and Adolescents

Seven cases (14%) developed symptoms before the age of 18. At the time of diagnosis or clinical description, all were adults. The time elapse between symptom onset and publication ranged from 9 years to approximately 70 years [9]. When age at onset was explicitly stated, the youngest reported was 15 years [14], although two additional cases were described as having childhood onset without a precise age given.

One individual being described with a probable childhood onset carried the common homozygous variant (NM_004130.4(*GYG1*): c.143+3G>C p.(?)), and initially presented with proximal muscle fatigability while running [4].

No evidence of cardiac involvement was identified: six cases had normal echocardiographic findings, and one was explicitly reported as asymptomatic with respect to cardiac symptoms.

### 4.6 Cardiac Presentation: Cardiomyopathy

Eight patients (16%) presented with cardiomyopathy, with age at onset ranging from 23 to 60 years. Hypertrophic cardiomyopathy, predominantly involving the left ventricle, was the most frequent cardiac phenotype (*n* = 6), while one patient presented with severe dilated cardiomyopathy [12]. One additional case was described as hypertensive heart disease without further characterization [14]. Cardiac MRI revealed myocardial scarring in two cases [5, 22]. All five available cardiac biopsies showed PAS‐positive, diastase‐resistant inclusions. Four patients required ICD implantation for arrhythmia prevention, and two underwent heart transplantation at ages 48 and 52 years [3, 5].

### 4.7 Cardiac and Muscular Presentation: Mixed Phenotype

Four cases presented both cardiomyopathy and muscular weakness. Aside from the youngest patient in this group, who received an ICD for secondary arrhythmia prevention (ventricular fibrillation, at 27 years [3]), none required ICD implantation or heart transplantation at time of data collection.

Four additional cases were diagnosed with coronary artery disease or had experienced a myocardial infarction, and three others were reported to have valvular disease, involving the tricuspid or mitral valves. One patient presented with a conduction defect, specifically a first‐degree atrioventricular block and left posterior hemiblock [22].

### 4.8 Phenotype–Genotype Correlation

All the cases had genetic confirmation with a homozygous or two compound heterozygous variants in *GYG1*.

Among the *GYG1 variant*s identified in the 49 cases, splice site variants were the most frequent, accounting for 46% of all the variants. A total of 61% of the variants were predicted to result in frameshift, and 22% were classified as missense variants. One patient carried a homozygous start loss variant c.2 T>A p.(?) [21].

The c.143+3G>C variant was identified 44 times, making it the most prevalent variant in the cohort. It was present in a homozygous state in 18 cases (36.7%). The second most common variant, c.304G>C p.(Asp102His), was observed 10 times with three homozygous cases.

Among the 18 homozygous cases for the c.143+3G>C variant, only one individual was reported to have cardiomyopathy, which was classified as hypertensive in nature and occurred alongside upper and lower limb weakness [14].

In this subgroup, the reported age of symptom onset ranged from childhood to 75 years, the latter observed in the present case. Eleven cases presented with weakness in both upper and lower limbs, four had isolated proximal lower limb weakness, and two exhibited both proximal and distal lower limb involvement. Scapular winging or weakness was noted in seven cases, and one patient developed facial weakness at 71 years of age [18]. This same patient became wheelchair‐dependent at 59 years. Notably, five of the six cases reported to require walking aids were carriers of the common *GYG1* c.143+3G>C variant. Asymmetric muscle weakness was described in three cases, while information on left–right distribution was unavailable in 14.

Supplemental findings: *GYG1* variants reported in literature and their reported frequency (Supplementary Tables 1 and 1A).

## 5 Discussion

Since the first description of glycogenin‐1 deficiency in 2010, fewer than 50 genetically confirmed cases have been reported, mainly as isolated case reports or small case series, highlighting the rarity and likely underdiagnosis of this condition [23]. In this study, we reviewed the clinical and genetic spectrum of 49 individuals with GYG1 variants and reported on the oldest known patient carrying the homozygous c.143+3G>C variant, with symptom onset at 75 years of age.

### 5.1 Clinical Presentation

GSD XV, caused by biallelic pathogenic variants in GYG1, predominantly presents as a skeletal muscle disease, with muscle weakness reported in 90% of cases. However, isolated cardiac involvement was observed in a minority of patients, supporting a broader clinical spectrum. Combined upper and lower limb weakness was the most frequent presentation, whereas isolated upper limb involvement was rare [4, 15].

Clinical severity and progression were highly variable. Although most patients remained ambulatory, 6 individuals required walking aids or wheelchairs, indicating that significant functional impairment may occur in a subset of patients [13, 18]. The distribution of weakness and associated features such as facial weakness or scapular winging was inconsistently reported, limiting conclusions regarding characteristic clinical patterns.

Age at onset ranged from childhood to late adulthood without clear genotype–phenotype correlation. This variability was particularly evident for the recurrent c.143+3G>C splice‐site variant, which was associated with symptom onset ranging from adolescence to 75 years in the present case [4]. Such variability may partly reflect residual normal splicing and variable glycogenin‐1 expression associated with this non‐canonical splice‐site variant, potentially influenced by splicing regulatory factors [13].

### 5.2 Diagnostic Challenges and Strategies

Before the first description of glycogenin‐1 deficiency by Moslemi et al. in 2010, many cases were misdiagnosed with limb‐girdle muscular dystrophy. Diagnostic delay remains common in GSD XV, particularly in patients with slowly progressive or atypical presentations, and many earlier cases were initially classified as other neuromuscular disorders prior to the identification of GYG1‐related disease. Although we did not formally stratify diagnostic delay by time period, more recent cases are increasingly diagnosed through next‐generation sequencing approaches, reflecting a shift from earlier biopsy‐driven diagnostic approaches [28].

Interestingly, previous authors have proposed a distinction between GSD XV, characterized predominantly by cardiomyopathy, and Polyglucosan Body Myopathy Type 2 (PGBM2), presenting mainly with skeletal muscle involvement based on the genotype [22]. Although our systematic review revealed substantial clinical variability among individuals with muscular involvement sharing the same genetic background [12, 13], the subgroup with isolated cardiac involvement is too small to draw conclusions regarding the aforementioned phenotypic distinction. The recurrent GYG1 variant c.143+3G>C was associated with a broad phenotypic spectrum ranging from early‐onset skeletal myopathy to late‐onset muscle disease with reported hypertensive heart disease [15]. Further investigation of this particular cardiac involvement is needed to clarify the potential contribution of the homozygous GYG1 variant to the development of heart disease. The collection of additional well‐characterized cases may help clarify whether GYG1‐related disease is better understood as a continuous phenotypic spectrum rather than two distinct clinical entities, with additional modifying mechanisms likely contributing to disease expression.

Before the association between *GYG1* gene and GSD XV, muscle biopsy remained the major diagnostic tool in diagnosing polyglucosan body disease. PAS‐positive, diastase‐resistant polyglucosan bodies are a hallmark of the disease under light microscopy. Although early reports described near absence of glycogen in muscle tissue, subsequent studies have demonstrated variable glycogen content, and this feature is not consistently observed. In our literature review, 33 biopsied cases showed PAS‐positive, diastase‐resistant inclusions consistent with polyglucosan bodies, whereas one report described PAS‐positive deposits without diastase resistance [19], suggesting that the absence of diastase resistance does not exclude the diagnosis.

The diagnostic value of additional EM analysis remains uncertain and needs further studies. This is illustrated by several cases in this cohort in which no identifiable polyglucosan bodies were detected on EM, although the absence was only explicitly stated by Akman et al.

Although most individuals (25/32) who underwent EMG or ENMG demonstrated a purely myopathic pattern, the presence of a neurogenic or mixed pattern should not exclude the diagnosis of GSD XV. In addition, mild neurogenic features such as fasciculations or fiber type grouping suggesting chronic reinnervation have been reported in some patients, including our case, supporting a broader neuromuscular spectrum rather than a purely myopathic process [4, 13, 14].

Genetic testing remains crucial as it allows for differentiation between the various forms of polyglucosan body diseases and helps exclude other types of glycogen storage disorders and cardiomyopathies. GYG1‐related disease is part of the differential diagnosis of polyglucosan body disorders, which include *GBE1* adult polyglucosan body disease (*GBE1*‐APBD) and RBCK1‐related polyglucosan body myopathy (*RBCK1*‐PGBM), all of which share PAS‐positive, diastase‐resistant glycogen inclusions [3, 23, 29, 30]. With the increasing availability of comprehensive gene panels for myopathies and cardiomyopathies, *GYG1* should be included systematically in these panels. In settings where genetic analysis is not immediately available, muscle biopsy findings can guide further work‐up. Functional validation of GYG1 variants remains limited, as no standardized assays are currently available in routine diagnostics; although research‐based approaches such as protein expression or autoglycosylation assays may provide supportive evidence, the lack of widely accessible functional testing represents a limitation, particularly for the interpretation of missense variants [2, 8].

### 5.3 The Role of Cardiac Evaluation and Surveillance

The eight cases of cardiomyopathy in this cohort highlight the potential for cardiac involvement in GSD XV, regardless of the genotype, and may distinguish GSD XV from other polyglucosan body diseases such as *GBE1‐*APBD [31], although the limited number of cases precludes definitive conclusions. On the other hand, severe dilated cardiomyopathy may raise the differential diagnosis of *RBCK1*‐PGBM with or without immunodeficiency [32]. While *RBCK1*‐PGBM generally presents in childhood, cardiomyopathy in GSD XV has, to date, only been reported in adults. Despite the limited cohort size, the occurrence of isolated or subclinical cardiac involvement supports cardiac evaluation and follow‐up, including echocardiography and cardiac MRI, even in cases presenting primarily with myopathic symptoms or carrying common GYG1 variants such as c.143+3G>C.

Cardiac manifestations may include hypertrophic or dilated cardiomyopathy, and recent reports, including two cases in our review, have described myocardial scarring or myocarditis‐like changes on cardiac MRI [5, 22]. Whether these findings represent a characteristic feature of GSD XV remains uncertain.

Cardiac biopsy remains highly invasive and has not shown additional diagnostic utility beyond genetic testing. It may nevertheless be considered in selected cases where the differential diagnosis remains uncertain, rather than as part of the routine evaluation of GSD XV.

### 5.4 Expanding the Phenotypic Spectrum

Mild memory loss and slightly reduced neurocognitive abilities were observed only in the present case. As cognitive impairment has not been reported in other individuals with GSD XV, these findings are more likely age‐related than disease‐specific, reinforcing the distinction from GBE1‐related adult polyglucosan body disease [31]. Mild self‐reported hearing loss in the present case was also described in two other cases [21, 24]. Except for two ischemic strokes, one occurring at 52 years, we did not find other atypical, previously unreported symptoms [20, 22]. No clear sex‐specific phenotype has emerged to date. Among the 20 cases with documented elevated CK levels, 8 were female and 12 were male, however, the available data do not allow a reliable assessment of sex‐related differences in CK levels.

### 5.5 Study Limitations

Our study is limited by the small number of published cases and the absence of large prospective cohorts. These limitations preclude definitive genotype–phenotype correlations. Moreover, genetic testing is not universally available or utilized in all clinical settings, particularly in older cases or those with non‐specific presentations. This may contribute to underdiagnosis or misclassification.

As GSD XV is an autosomal recessive condition, it often lacks a suggestive family history, further delaying diagnosis. In most reports, cascade testing among siblings or extended family members has not been performed or documented, making it difficult to assess incomplete penetrance or detect asymptomatic carriers.

Another major challenge is the current classification of genetic variants. While several coding variants remain of uncertain significance, in cases where one or both GYG1 variants are located in a deep intronic region, genome sequencing is essential but remains unavailable in many clinical settings.

Lastly, an important limitation of our study was the heterogeneity of the included studies and case reports. In some cases, it was unclear whether certain symptoms or analytic criteria were unavailable or absent, complicating data interpretation.

### 5.6 Perspectives and Future Directions

Further case reports and prospective registries are essential to better define the full clinical spectrum of individuals with GYG1 variants, including early cardiac changes, potential sex differences, and genotype‐specific risks. Longitudinal follow‐up of both symptomatic and asymptomatic individuals with GYG1‐associated conditions will be crucial in determining disease progression and optimizing surveillance strategies.

In conclusion, while individuals with GYG1 variants predominantly present with a skeletal muscle disease, occasional neurogenic features and cardiac manifestations may occur in isolation or as part of a combined phenotype. Because clinical presentation is highly variable and frequently overlaps with other myopathies or polyglucosan body diseases, early genetic testing combined with targeted histopathological evaluation is essential for diagnosis. Broader inclusion of GYG1 in neuromuscular and cardiomyopathy gene panels may help reduce underdiagnosis and improve clinical surveillance.

## Author Contributions

M.S. and C.T. conceived and designed the study. J.E. developed search strategy. M.S. and A.T. performed literature review and article selection. M.S. performed data extraction, statistical calculations and manuscript draft. C.T. supervised the data extraction and writing process. E.H. provided the histological figures. F.M. did sequence analysis for the case report. P.M. conducted cardiac evaluation and M.T. performed neurological investigations. All authors critically revised it for important intellectual content. All authors approved the final version of the manuscript.

## Funding

The authors have nothing to report.

## Ethics Statement

Ethical review and approval were not required for the study on human participants in accordance with the local legislation and institutional requirements.

## Consent

The patient provided his written consent to participate in this publication.

## Conflicts of Interest

The authors declare no conflicts of interest.

## Supporting information


**Supplementary Figure 1.** PRISMA 2020 flow diagram for new systematic reviews which included searches of databases, registers, and other sources.

**Supplementary Table 1.** *GYG1* sequence variants.

**Supplementary Table 1A.** Frequency of the reported variants.

## Data Availability

The data that support the findings of this study are available from the corresponding author upon reasonable request.
