# Supplementary material for: Genotype and Phenotype Description in Individuals With GYG1 Variants—A Case Report and Systematic Review
Source: JIMD Rep. 2026 Nov 5;67(6):e70134. doi: 10.1002/jmd2.70134 (PMC13737462; doi:10.1002/jmd2.70134)
Supplement: Supplementary file 1 [file JMD2-67-e70134-s001.docx]

**Supplementary Table 1. *GYG1* sequence variants**

In blue: most common variant: c.143+3G>C p.(?)

**^#^**Deep intronic genomic variant reported as originally described by Fanin et al.
