# Supplementary material for: Genotype and Phenotype Description in Individuals With GYG1 Variants—A Case Report and Systematic Review
Source: JIMD Rep. 2026 Nov 5;67(6):e70134. doi: 10.1002/jmd2.70134 (PMC13737462; doi:10.1002/jmd2.70134)
Supplement: Supplementary file 2 [file JMD2-67-e70134-s003.docx]

**Identification of studies via other methods**

**Identification of studies via databases and registers**

Records removed *before screening*:

Duplicate records removed with Deduklick (n = 38)

Duplicate records removed manually (n = 3)

Records removed for other reasons (n = 4)

Records identified from*:

MEDLINE (n = 22)

Embase (n = 32)

Cochrane CENTRAL (n = 2)

Web of Science (n = 18)

PMC Central (n = 16)

ClinicalTrials.gov (n = 3)

ICTRP (n = 2)

Google Scholar (n = 25)

TOTAL (n = 120)

Records identified from:

Websites (n = 4)

PMC Central (n= 1)

Pubmed (n=3)

**Identification**

Records screened

(n = 75)

Records excluded**

(n = 48)

Reports not retrieved

(n = 0)

Reports sought for retrieval

(n = 4)

Reports sought for retrieval

(n = 48)

Reports not retrieved

(n = 14)

**Screening**

Reports excluded:

Absence of relevant clinical data (n = 12)

Previously reported case (n = 6)

Reports assessed for eligibility

(n = 4)

Reports assessed for eligibility

(n = 35)

Reports of included studies

(n = 17)

**Included**

Studies included in review

(n = 21)

Reports of included studies

(n = 21)

Reports of included studies

(n = 4)

*Consider, if feasible to do so, reporting the number of records identified from each database or register searched (rather than the total number across all databases/registers).

**If automation tools were used, indicate how many records were excluded by a human and how many were excluded by automation tools.

*From:*  Page MJ, McKenzie JE, Bossuyt PM, Boutron I, Hoffmann TC, Mulrow CD, et al. The PRISMA 2020 statement: an updated guideline for reporting systematic reviews. BMJ 2021;372:n71. doi: 10.1136/bmj.n71. For more information, visit: <http://www.prisma-statement.org/>
